# Multi-branch GAT-GRU-transformer for explainable EEG-based finger motor imagery classification

**DOI:** 10.3389/fnhum.2025.1599960

**Published:** 2025-05-21

**Authors:** Zhuozheng Wang, Yunlong Wang

**Affiliations:** Faculty of Information Technology, Beijing University of Technology, Beijing, China

**Keywords:** finger motor imagery classification, EEG, multi-branch, deep learning, explainability

## Abstract

Electroencephalography (EEG) provides a non-invasive and real-time approach to decoding motor imagery (MI) tasks, such as finger movements, offering significant potential for brain-computer interface (BCI) applications. However, due to the complex, noisy, and non-stationary nature of EEG signals, traditional classification methods—such as Common Spatial Pattern (CSP) and Power Spectral Density (PSD)—struggle to extract meaningful, multidimensional features. While deep learning models like CNNs and RNNs have shown promise, they often focus on single-dimensional aspects and lack interpretability, limiting their neuroscientific relevance. This study proposes a novel multi-branch deep learning framework, termed Multi-Branch GAT-GRU-Transformer, to enhance EEG-based MI classification. The model consists of parallel branches to extract spatial, temporal, and frequency features: a Graph Attention Network (GAT) models spatial relationships among EEG channels, a hybrid Gated Recurrent Unit (GRU) and Transformer module captures temporal dependencies, and one-dimensional CNNs extract frequency-specific information. Feature fusion is employed to integrate these heterogeneous representations. To improve interpretability, the model incorporates SHAP (SHapley Additive exPlanations) and Phase Locking Value (PLV) analyses. Notably, PLV is also used to construct the GAT adjacency matrix, embedding biologically-informed spatial priors into the learning process. The proposed model was evaluated on the Kaya dataset, achieving a five-class MI classification accuracy of 55.76%. Ablation studies confirmed the effectiveness of each architectural component. Furthermore, SHAP and PLV analyses identified C3 and C4 as critical EEG channels and highlighted the Beta frequency band as highly relevant, aligning with known motor-related neural activity. The Multi-Branch GAT-GRU-Transformer effectively addresses key challenges in EEG-based MI classification by integrating domain-relevant spatial, temporal, and frequency features, while enhancing model interpretability through biologically grounded mechanisms. This work not only improves classification performance but also provides a transparent framework for neuroscientific investigation, with broad implications for BCI development and cognitive neuroscience research.

## 1 Introduction

Electroencephalography (EEG) is a non-invasive, real-time, and cost-effective neurophysiological monitoring technique that records the electrical activity of the cerebral cortex, revealing neural dynamic patterns associated with motor intentions, such as finger or limb movements (Niedermeyer and da Silva, 2005). In recent years, EEG signals have shown increasing potential in applications such as Brain-Computer Interfaces (BCIs), neurorehabilitation, and intelligent assistive devices, enabling advancements in motor recovery for stroke patients, control of prosthetic limbs, and communication for individuals with severe motor disabilities (McFarland, 2002; Pfurtscheller et al., 2008). However, the high noise, non-stationarity, and complex spatio-temporal-frequency characteristics of EEG signals make accurate motor intention recognition, particularly for fine-grained tasks like finger movements, a highly challenging task. These challenges are compounded by inter-subject variability and the low signal-to-noise ratio of EEG data, which often lead to inconsistent performance across different datasets and subjects (Blankertz et al., 2010).

EEG signal processing for motor intention recognition has been extensively explored, encompassing both traditional and deep learning approaches, with notable performance advancements. Traditional methods primarily rely on handcrafted feature extraction and shallow classifiers. For instance, Common Spatial Pattern (CSP) extracts spatial features by maximizing inter-class variance and is often paired with classifiers like Support Vector Machines (SVM), achieving robust performance in binary motor imagery tasks (Ramoser et al., 2000). Power Spectral Density (PSD) analysis computes the power of EEG signals across different frequency bands (e.g., Alpha: 8–13 Hz, Beta: 13–30 Hz) to derive frequency-domain features linked to motor intentions (Zhang et al., 2020). Extensions like Filter Bank CSP (FBCSP) further improve performance by analyzing multiple frequency bands, achieving better feature discrimination in multi-class settings, such as the BCI Competition IV datasets (Ang et al., 2012). Other traditional methods, such as Wavelet Transform (WT), decompose EEG signals into time-frequency representations to capture transient features associated with motor events (Herman et al., 2008). Despite their effectiveness in specific contexts, these methods require intricate preprocessing and feature engineering, and their performance diminishes when confronted with high-noise, non-stationary EEG signals. Notably, they struggle to integrate multidimensional information across spatial (inter-channel dependencies), temporal (dynamic evolution), and frequency (specific brain rhythms) domains, resulting in limited classification efficacy for complex tasks like five-finger motor imagery, where subtle differences in EEG patterns are critical (Pfurtscheller and Lopes da Silva, 1999).

The advent of deep learning has introduced transformative solutions to EEG classification tasks, mitigating some of the limitations of traditional methods by automating feature extraction. Convolutional Neural Networks (CNNs), such as EEGNet, are widely adopted for their proficiency in extracting local spatial and frequency patterns, achieving competitive accuracies like 51.73% on the Kaya dataset for five-finger motor imagery tasks (Lawhern et al., 2018). Recurrent Neural Networks (RNNs), along with their variants such as Long Short-Term Memory (LSTM) units and Gated Recurrent Units (GRU), excel at modeling temporal dependencies within EEG signals, making them suitable for capturing the dynamic evolution of motor-related brain activity (Zheng et al., 2017). Hybrid models like CNN-LSTM combine spatial and temporal feature extraction, further improving performance in motor imagery tasks by achieving accuracies up to 48–50% on similar datasets (Zhao et al., 2019). Moreover, Transformer models, leveraging self-attention mechanisms, have demonstrated exceptional capability in capturing long-range temporal dependencies, finding successful application in EEG classification tasks with reported improvements in multi-class settings (Tan et al., 2023). Recent advancements also include transfer learning and domain adaptation techniques, which aim to address inter-subject variability by pre-training models on large EEG datasets and fine-tuning them on target subjects, as seen in works like DeepConvNet (Schirrmeister et al., 2017) and cross-subject transfer learning frameworks (Fahimi et al., 2020). Despite these advances, most deep learning methods focus on single-dimensional features—CNNs on spatial or frequency patterns, and RNNs or Transformers on temporal dynamics—making it difficult to fully represent the multi-dimensional spatio-temporal-frequency nature of EEG signals. Additionally, the opaque “black-box” nature of these models limits their interpretability, obscuring the neurophysiological mechanisms underlying their decisions and restricting their utility in clinical diagnostics and neuroscience research, where understanding the neural basis of predictions is crucial (Lotte et al., 2018). Furthermore, transfer learning approaches often struggle with domain shifts between subjects, leading to reduced generalization performance in fine-grained tasks.

The inherent inter-channel relationships in EEG signals naturally lend themselves to graph-based modeling, positioning Graph Neural Networks (GNNs) as a burgeoning research focus. Graph Attention Networks (GATs), in particular, enhance spatial relationship modeling by dynamically learning the significance of inter-channel connections via attention mechanisms, offering a more adaptive approach compared to traditional GNNs (Velickovic et al., 2017). GATs have outperformed traditional GNNs in tasks such as emotion recognition, achieving accuracies up to 85% on public datasets (Zhong et al., 2020), and epilepsy detection, where they improved seizure prediction by 5–10% over baseline methods (Jibon et al., 2024). More recent studies have explored multi-layer GAT architectures to capture hierarchical spatial patterns, such as local interactions between nearby electrodes and global connectivity across brain regions (Klepl et al., 2024). However, existing studies predominantly employ single-layer GAT architectures, which fail to fully leverage the hierarchical spatial structure of EEG signals and encounter challenges related to complex adjacency matrix design and computational inefficiency, particularly when scaling to larger datasets (Almohammadi, 2024). Additionally, the integration of GATs with temporal and frequency feature extraction remains underexplored, limiting their ability to address the multidimensional nature of EEG data (Liu et al., 2024).

To mitigate the “black-box” limitation of deep learning models, interpretability techniques have emerged as valuable tools for understanding model decisions in EEG classification. SHAP (SHapley Additive exPlanations) quantifies the contribution of input features to model predictions, pinpointing critical channels (e.g., C3, C4) and time segments in classification tasks, which has been applied to motor imagery studies to identify key EEG features (Lundberg and Lee, 2017; Zhou et al., 2023). Similarly, Phase Locking Value (PLV) evaluates phase synchrony between EEG channels, shedding light on functional connectivity across brain regions, such as increased connectivity in the Beta band during motor tasks (Lachaux et al., 1999). Other interpretability methods, such as Grad-CAM, have been used to visualize the spatial focus of CNN models in EEG classification, highlighting active brain regions (Selvaraju et al., 2017). While these methods offer partial insights, few studies integrate SHAP and PLV to concurrently assess feature importance and neural connectivity, leaving interpretations of EEG signal processing models incomplete (Rajpura et al., 2024). Moreover, the application of interpretability techniques in fine-grained tasks like five-finger motor imagery remains limited, where understanding subtle neural differences is essential for advancing neuroscientific knowledge.

Despite significant strides in EEG signal processing, current methods exhibit critical shortcomings that highlight the need for this study. Traditional approaches like CSP, PSD, and FBCSP, as well as most deep learning models, emphasize single-dimensional modeling—focusing solely on spatial, temporal, or frequency features—failing to capture the full multi-dimensional essence of EEG signals. Spatial modeling remains inadequate, as existing GAT applications are often confined to single-layer structures, underutilizing the hierarchical spatial information inherent in EEG data. Moreover, while interpretability methods like SHAP, PLV, and Grad-CAM provide some clarity, their rare integration with multi-dimensional feature extraction frameworks limits a comprehensive understanding of model behavior, particularly for fine-grained tasks like five-finger motor imagery classification, where subtle EEG pattern differences pose significant challenges. Additionally, transfer learning and domain adaptation methods, while promising, struggle to generalize across subjects in such tasks due to domain shifts, often achieving accuracies below 50% in cross-subject settings.

To address these deficiencies, this paper introduces the “Multi-Branch GAT-GRU-Transformer,” a multi-branch deep learning model with innovative designs. The model features three parallel branches: one employing Graph Attention Networks (GAT) to model spatial relationships, another combining GRU and Transformer to capture temporal dependencies, and a third utilizing one-dimensional Convolutional Neural Networks (1D CNN) to extract frequency features, thereby achieving a holistic representation of EEG signals’ multi-dimensional characteristics. In the spatial branch, a hierarchical GAT structure is implemented, where lower layers learn local inter-channel interactions, and higher layers capture global spatial patterns across brain regions, enhancing spatial feature extraction. The temporal branch integrates GRU for local temporal dependency modeling with a Transformer Encoder for long-range dependencies via self-attention, augmented by a temporal attention mechanism to heighten sensitivity to pivotal time points. In the frequency branch, multi-band filtering separates EEG signals into distinct frequency bands (e.g., Delta, Theta, Alpha, Beta, Gamma), with 1D CNN extracting band-specific features pertinent to motor intentions. An interpretability module further enhances the model by employing SHAP to quantify the contributions of input EEG signals to decisions—identifying key channels, time segments, and frequency bands—while PLV analyzes inter-channel phase synchrony to reveal cooperative brain region activity under varying motor intentions.

The primary contributions of this work are threefold. First, it proposes a multi-branch model integrating GAT, GRU, Transformer, and 1D CNN, achieving a test accuracy of 55.76% on the Kaya dataset, outperforming baselines like EEGNet (51.73%). Second, it incorporates SHAP and PLV techniques for interpretability analysis, offering novel scientific insights into the neural mechanisms of finger motor intentions, such as the reliance on C3, C4, and Cz in Beta and Gamma bands. Third, it validates the model’s superior performance through statistical significance tests and provides a deeper understanding of brain activity patterns through interpretability analysis. Through these advancements, this study not only elevates the technical sophistication of EEG signal processing but also provides new methodologies and perspectives for research in brain-computer interfaces and neuroscience.

## 2 Materials and methods

### 2.1 Dataset

The experiment utilizes the large-scale EEG motor imagery dataset provided by Kaya et al. (2018), comprising EEG recordings from 13 healthy participants (8 males and 5 females) aged between 20 and 35 years. The dataset adopts a five-finger motor imagery (5F) paradigm, wherein participants engage in motor imagery tasks involving individual fingers: the thumb, index, middle, ring, and little fingers. EEG signals were recorded using the BrainAmp system (Brain Products, Germany) at a sampling rate of 1,000 Hz, ensuring high temporal resolution necessary for capturing transient neural dynamics. A total of 22 EEG channels were employed, selected in accordance with the international 10–20 system. This standardized electrode placement scheme determines scalp locations based on proportional distances between key anatomical landmarks, ensuring consistent and reproducible electrode positioning across subjects. The use of this system is particularly advantageous for its robust coverage of functionally significant cortical areas, notably the motor cortex regions (e.g., channels C3, C4, and Cz), which are critical for decoding motor-related brain activity. The 5F paradigm consists of five distinct classes, each corresponding to the imagery of a specific finger’s movement, resulting in approximately 15,000 trials in total, with an average of 1,154 samples per subject. Each trial spans 4 s, during which participants are instructed to imagine the movement of a designated finger in response to visual cues. This well-structured experimental protocol enables a fine-grained analysis of cortical activations associated with individual finger motor imagery, thereby supporting high-resolution decoding tasks in EEG-based brain-computer interface research.

### 2.2 Preprocessing

To improve EEG signal quality and minimize noise interference, the experiment incorporates a series of preprocessing steps designed to ensure signal cleanliness and the accuracy of subsequent analyses.

To enhance EEG signal quality and minimize noise interference, the experiment employs a comprehensive preprocessing pipeline to ensure signal cleanliness and the accuracy of subsequent analyses. First, a 4th-order Butterworth bandpass filter (0.5–100 Hz) is applied to retain frequency bands relevant to motor intentions while eliminating low-frequency drift and high-frequency noise. This range encompasses Delta, Theta, Alpha, Beta, and portions of Gamma waves, all associated with motor planning and execution. Next, the signals are resampled from 1,000 Hz to 128 Hz using interpolation and decimation techniques, reducing computational complexity while preserving essential motor-related features. To further refine signal quality, Independent Component Analysis (ICA) is used to remove ocular and muscular artifacts. The raw EEG signals undergo ICA decomposition, and an automatic detection algorithm identifies and eliminates components associated with eye movements and muscle activity (Bigdely-Shamlo et al., 2015). The cleaned EEG signals are then reconstructed, ensuring minimal interference from non-neural sources. Following artifact removal, continuous EEG signals are segmented into 2-s epochs, beginning 0.5 s before and ending 1.5 s after the motor intention trigger, effectively capturing both preparation and execution phases. Finally, Z-score normalization is applied to each channel by subtracting the mean and dividing by the standard deviation, eliminating amplitude variations across channels due to electrode contact differences or individual variability (Pedroni et al., 2019). This normalization step standardizes the signals, improving model training stability and feature comparability.

### 2.3 Model architecture

**Figure 1:**
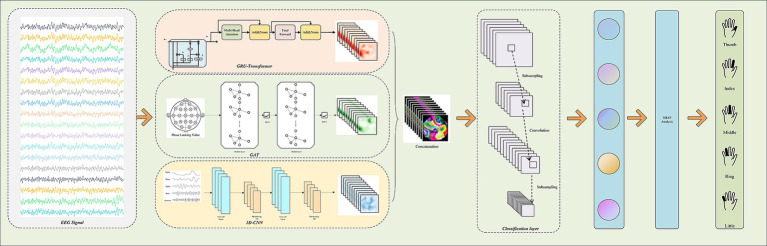
Architecture and detailed design of the proposed multi-branch GAT-GRU-transformer model for EEG-based five-finger motor imagery classification.

**Figure 2:**
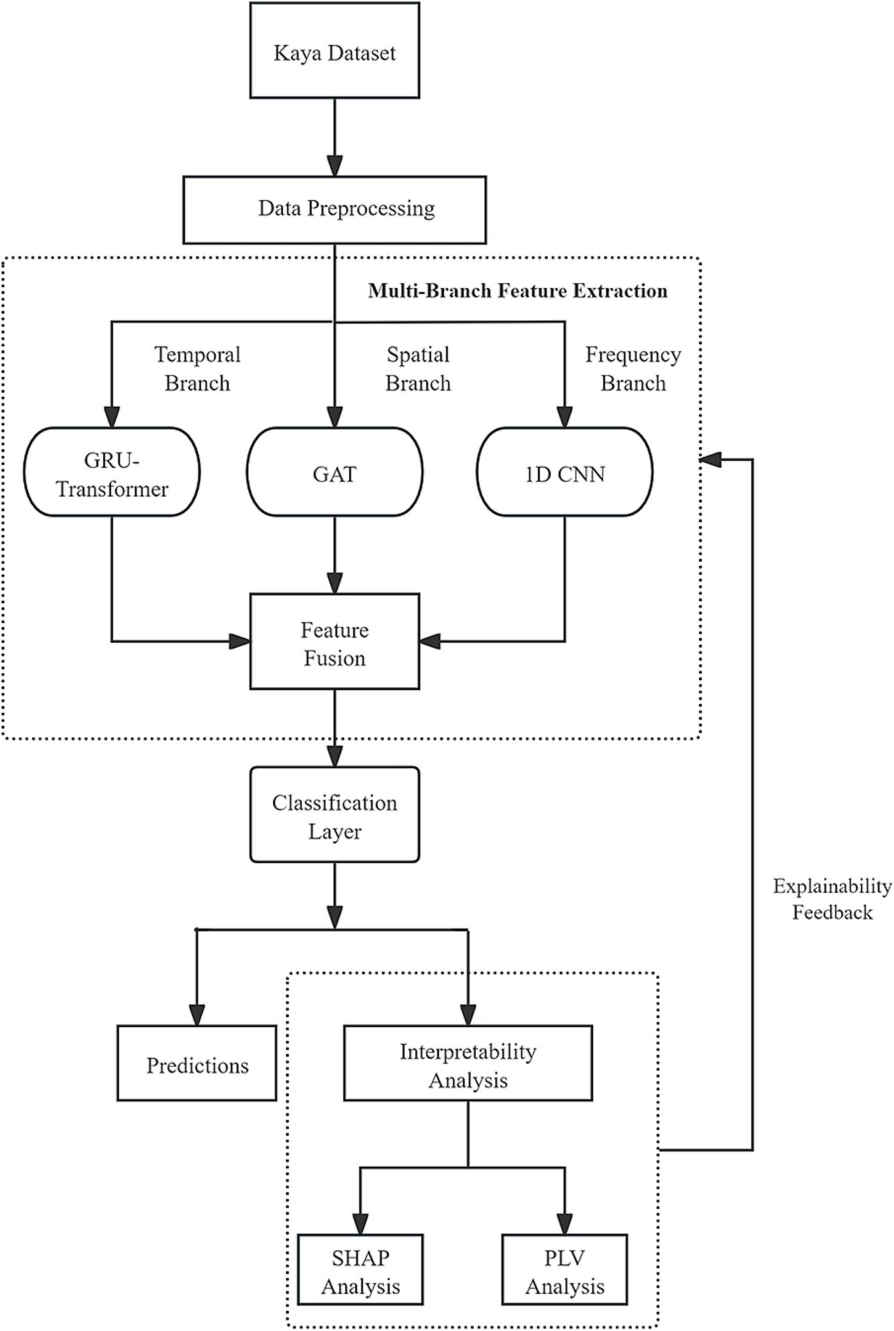
Overview of the proposed multi-branch GAT-GRU-transformer framework for EEG-based finger motor imagery classification.

To comprehensively capture the spatial, temporal, and frequency features of EEG signals and to provide a scientific explanation for the model’s decision-making process, this paper proposes a novel multi-branch deep learning model named “Multi-Branch GAT-GRU-Transformer.” This model processes different dimensional features of EEG signals in parallel, integrates these features for classification, and incorporates interpretability techniques to elucidate the underlying decision-making mechanism. Figure 1 illustrates the overall architecture of the model. The model comprises three primary branches:Spatial Modeling Branch (GAT) utilizes a Graph Attention Network (GAT) to capture spatial relationships between EEG channels. Temporal Modeling Branch (GRU + Transformer) extracts temporal features from EEG signals using a Gated Recurrent Unit (GRU) combined with a Transformer Encoder. Frequency Modeling Branch (1D CNN) employs a one-dimensional Convolutional Neural Network (1D CNN) to extract frequency-domain features from multi-band filtered EEG signals. The output features from each branch are integrated via a multimodal fusion module, with the final classification task performed using a CNN. Additionally, the model includes an interpretability module that leverages SHAP techniques to analyze the neural basis of the model’s decisions. The overall workflow is illustrated in Figure 2.

#### 2.3.1 Spatial modeling (GAT branch)

The spatial characteristics of EEG signals reflect the relationships of electrical activity across different brain regions. To capture spatial interactions between EEG channels, this model adopts a Graph Attention Network (GAT) for spatial modeling. The GAT was chosen for spatial feature extraction due to its ability to dynamically learn inter-channel dependencies, which is critical for capturing the spatial dynamics of EEG signals during motor imagery tasks. Unlike traditional graph-based methods, which provide a static adjacency matrix based on phase synchrony, GAT employs a self-attention mechanism to assign learnable attention coefficients, enabling adaptive spatial modeling tailored to the task. Furthermore, the hierarchical GAT structure in our framework captures both local and global spatial dependencies, enhancing feature extraction. This adaptability and hierarchical abstraction contribute to improved noise robustness and classification performance. Through its graph structure and attention mechanisms, GAT dynamically learns the importance of relationships between channels, thereby extracting rich spatial features (Demir et al., 2022). The performance of GAT depends on the design of the graph’s adjacency matrix, which defines the connectivity relationships between EEG channels. Using spatial position information of EEG electrodes, connections are established according to the physical distance between electrodes. If the distance between two electrodes is less than a preset threshold *d*, they are connected in the adjacency matrix with an edge weight of 1; otherwise, the edge weight is set to 0. This method capitalizes on the correlation of functional activities between adjacent areas of the cerebral cortex, making it well-suited for capturing local spatial dependencies (Li et al., 2023). In practical applications, to construct the graph for the GAT in the spatial branch, PLV is computed once across frequency bands to quantify inter-channel phase synchrony, serving as a biologically inspired prior for the adjacency matrix. By providing a neurophysiologically grounded graph structure, PLV enhances the efficiency of the GAT’s spatial feature extraction, as reflected in the model’s improved performance. This design not only preserves prior knowledge of spatial positions but also reduces computational complexity through sparsity, preventing unnecessary connections from interfering with feature extraction.

To extract multi-scale spatial features, we designed a hierarchical Graph Attention Network (GAT) structure. Low-level GAT consists of multiple GAT layers, where each layer learns local interactions between EEG channels through an attention mechanism. The number of attention heads is set to 4–8 to capture multi-dimensional local spatial patterns. The output of the low-level GAT is local spatial features as shown in Equation 1:


(1) $$H_{\mathrm{low}}\in R^{C\times D_{\mathrm{low}}}$$

Where C represents the number of channels and Dlow is the feature dimension. This module focuses on local interactions between adjacent channels. High-level GAT building on the output of the low-level GAT, additional GAT layers are employed to learn global spatial patterns across brain regions. The attention mechanism in the high-level GAT integrates local features from different brain areas, capturing broader inter-channel dependencies. The output is global spatial features as shown in Equation 2:


(2) $$F_{\text{spatial}}\in R^{C\times T\times D}$$

Where C is the number of channels, T is the number of time points, and D is the feature dimension (Zhao et al., 2021).

The hierarchical GAT design enables the model to extract spatial information at different scales: the low-level GAT focuses on local interactions, while the high-level GAT emphasizes global patterns. This hierarchical processing significantly enhances the model’s ability to represent the spatial characteristics of EEG signals (Cisotto et al., 2020).

#### 2.3.2 Temporal modeling (GRU + transformer branch)

Temporal dependencies in EEG signals are critical for motor intention recognition. To address this, we designed a module combining a Gated Recurrent Unit (GRU) with a Transformer Encoder to capture both local and long-range temporal dependencies. As a variant of Recurrent Neural Networks (RNN), the GRU effectively processes the short-term dynamics of EEG signals. The GRU layer receives the raw EEG signal A unidirectional GRU is adopted, capturing local temporal dependencies through gating mechanisms (Chen et al., 2019). The number of hidden units is set to 128 to balance model capacity and computational efficiency. The output is local temporal features as shown in Equation 3


(3) $$\begin{array}{c} H_{\mathrm{GRU}}\in R^{C\times T\times D\prime } \end{array}$$

Where D′ is the feature dimension of the GRU hidden layer (Xu et al., 2023). Through pointwise processing of the time series, the GRU layer extracts short-term dynamic patterns from the EEG signal, providing foundational features for the subsequent Transformer Encoder.

To capture long-range temporal dependencies, a Transformer Encoder is incorporated following the GRU layer. The Transformer Encoder computes relationships between time points using a self-attention mechanism and introduces a temporal attention mechanism to enhance sensitivity to critical time segments: Self-Attention Mechanism calculates the relationships between different time points, enabling the model to capture long-range dependencies (Sun et al., 2021). Learnable temporal embedding vectors are introduced to dynamically weight the importance of different time points, improving the model’s sensitivity to key temporal segments. Each sub-layer in the Transformer Encoder is followed by a feed-forward network for nonlinear transformation and feature enhancement (Song et al., 2022). The output of the Transformer Encoder is temporal features as shown in Equation 4:


(4) $$\begin{array}{c} F_{\text{time}}\in R^{C\times T\times D\prime \prime } \end{array}$$

Where *D″* is the output dimension of the Transformer Encoder.

The combination of GRU and Transformer for temporal feature extraction leverages the strengths of both architectures to model the complex temporal dynamics of EEG signals. GRU captures local and short-term dependencies, such as event-related synchronization (ERS) and desynchronization (ERD), while the Transformer’s self-attention mechanism models global temporal dependencies across the entire sequence, identifying long-range patterns critical for motor imagery tasks. This hybrid structure, augmented by a temporal attention mechanism, facilitates a richer temporal representation, enhances interpretability by focusing on key time segments, and improves classification performance.

#### 2.3.3 Frequency modeling (1D CNN branch)

The frequency-domain features of EEG signals are closely tied to motor intentions, with different frequency bands reflecting various brain activity states. To this end, we designed a multi-band 1D Convolutional Neural Network (CNN) module to extract features from EEG signals across different frequency bands (Ma et al., 2021). First, the EEG signal is decomposed into multiple physiological frequency bands using band-pass filters, including: Delta (0.5–4 Hz), Theta (4–8 Hz), Alpha (8–13 Hz), Beta (13–30 Hz), Gamma (30–100 Hz). These frequency bands are selected based on neuroscience research and encompass EEG activities related to motor intentions.

For each frequency band, a one-dimensional Convolutional Neural Network (1D CNN) is independently applied to extract local patterns. Each frequency band’s 1D CNN comprises multiple convolutional layers with kernel sizes of 3–5, a stride of 1, and ReLU activation functions to extract local frequency features. Max pooling layers follow the convolutional layers to reduce feature dimensions and enhance translation invariance (Altıntop et al., 2022). The CNN output features from each frequency band are fused through concatenation, yielding frequency features as shown in Equation 5:


(5) $$\begin{array}{c} F_{\text{freq}}\in R^{C\times T\times D\prime \prime \prime } \end{array}$$

Where *D″*′ is the fused feature dimension. Through the multi-band 1D CNN, the model extracts specific features from EEG signals in different frequency bands and achieves a comprehensive representation of frequency information via fusion.

### 2.4 Multimodal fusion

To effectively integrate features from the spatial (GAT branch), temporal (GRU + Transformer branch), and frequency (1D CNN branch) modalities, we designed a multimodal fusion module that incorporates a fusion mechanism to dynamically combine the importance of each modality (Deligani et al., 2021). This approach not only enhances the model’s classification performance but also improves its adaptability to different tasks and samples, while supporting subsequent interpretability analysis. Initially, the output features from the three branches are concatenated to generate a comprehensive feature representation as shown in Equation 6:


(6) $$\begin{array}{c} F_{\text{concat}}=C\text{oncat}(F_{\text{spatial}},F_{\text{time}},F_{\text{freq}})\in R^{C\times T\times (D+D\prime \prime +D\prime \prime \prime )} \end{array}$$

Where $F_{\text{spatial}}$ ∈ $R^{C\times T\times D}$ denotes spatial features, $F_{\text{time}}$ ∈ RC×T×D″ denotes temporal features, and $F_{\text{freq}}$ ∈ $R^{C\times T\times D\prime \prime }$ denotes frequency features, with *C* being the number of channels, *T* the number of time steps, and *D′, D″, D″*′ the respective feature dimensions of each branch.

To reduce dimensionality and computational complexity, Global Average Pooling is applied to *F_concat_*, resulting in a compact feature vector as shown in Equation 7.


(7) $$\begin{array}{c} F_{\text{pool}}=\mathrm{GAP}(F_{\text{concat}})\in R^{D+D\prime \prime +D\prime \prime \prime } \end{array}$$

This pooling operation retains key information while significantly reducing the parameter count, thereby improving training efficiency (Cai et al., 2020).

### 2.5 Interpretability module

To elucidate the neural basis of the model’s decision-making in the EEG signal motor intention recognition task and enhance its transparency and trustworthiness, we designed an interpretability module combining SHAP (SHapley Additive exPlanations) and PLV (Phase Locking Value) techniques. These methods analyze the model’s decision-making process from different perspectives: SHAP quantifies the contribution of each input feature to the model’s output, identifying the most critical EEG signal features for classification; PLV evaluates phase synchronization patterns between EEG channels, revealing cooperative activity and functional connectivity across brain regions. By integrating these techniques, we clarify the EEG features the model focuses on and gain deeper insights into the neural mechanisms underlying finger motor intentions, supporting the model’s scientific interpretation and application.

#### 2.5.1 SHAP analysis in EEG-based classification

**Figure 3:**
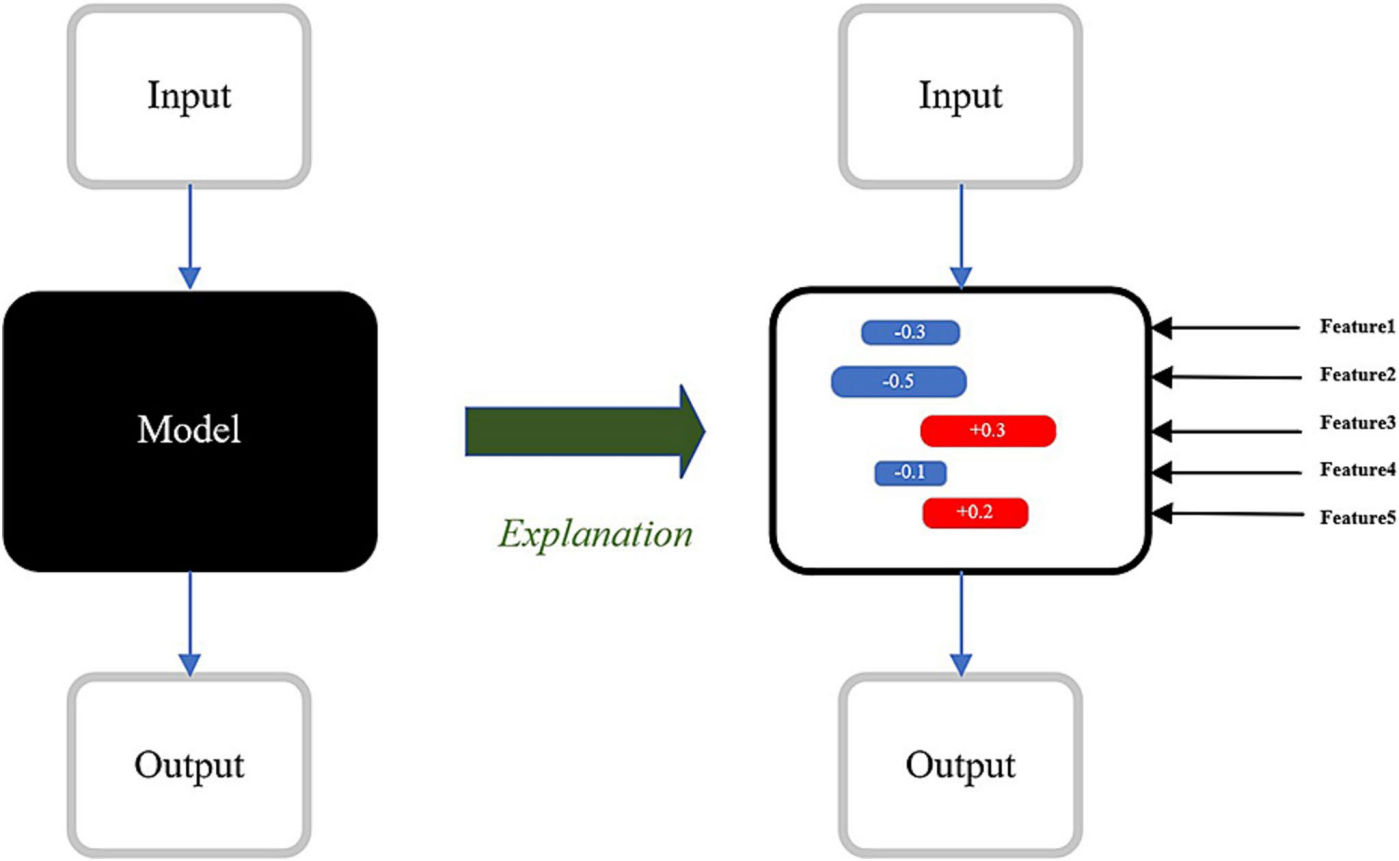
SHAP mechanism of action.

SHAP, a feature importance analysis method based on cooperative game theory, quantifies the contribution of input EEG signals to the model’s predictions as shown in Figure 3 (Raab et al., 2023). In this project, SHAP is applied to the multi-branch model’s output to interpret its reliance on specific features for classifying finger motor intentions.

For each EEG sample, SHAP values are computed for each channel, time point, and frequency band. These values quantify the positive or negative contribution of specific features to the model’s prediction of finger motor intentions.

By summarizing and ranking SHAP values, the most influential EEG channels, time segments, and frequency bands for the classification task can be identified. In motor intention recognition, the model may exhibit higher SHAP values over channels associated with the motor cortex, particularly within critical time windows following the onset of motor intention.

SHAP visualization tools provide an intuitive presentation of the distribution patterns of key features. A heatmap is used to show the distribution of SHAP values across different channels and time points, highlighting the brain regions and temporal dynamics that the model focuses on (Khare and Acharya, 2023). Additionally, a bar graph is employed to display the average SHAP values for different frequency bands, revealing the model’s reliance on specific frequency components.

Through SHAP analysis, it is possible to identify that the model’s decisions are highly consistent with known phenomena in neuroscience, thereby enhancing the interpretability and scientific credibility of the model.

#### 2.5.2 PLV analysis in EEG-based classification

Phase Locking Value (PLV) is a phase synchronization analysis technique used to evaluate the coordinated activity between EEG channels, reflecting functional connectivity between brain regions. In this study, PLV is employed to investigate the neural mechanisms underlying the model’s decisions, particularly the dynamic changes in brain networks during finger motor intention tasks. PLV values are computed between EEG channels within specific frequency bands (Chouhan et al., 2018). PLV quantifies the degree of synchronization by measuring the stability of the phase difference between two signals, with values ranging from 0 (completely desynchronized) to 1 (fully synchronized). In finger motor intention tasks, channel pairs with higher PLV values may reflect cooperative activity between the motor cortex and supplementary motor areas.

In the interpretability module, PLV is used to quantify functional connectivity between EEG channels, complementing SHAP’s feature attribution analysis. By measuring phase synchrony, PLV validates the spatial relationships learned by the GAT, confirming that high-attention channels (e.g., C3, C4) exhibit strong connectivity in the Beta band, consistent with motor imagery processes. This dual-level interpretability enhances our understanding of the model’s decisions and their neurophysiological basis. By identifying channel pairs with high PLV values, the functional connectivity patterns between different brain regions can be revealed. Significant phase synchronization between the motor cortex and the prefrontal or supplementary motor areas may indicate coordinated activity involved in motor planning and execution. This pattern aligns with existing neuroscience research on brain networks related to motor intention. The PLV is calculated using the Equation 8:


(8) $$\begin{array}{c} \mathrm{PLV}=|\frac{1}{T}\sum_{t=1}^{T}e^{\mathrm{i\Delta \Phi}(t)}| \end{array}$$

Where T is the total number of time points, and ΔΦ(t) is the phase difference at time point t. $e^{\mathrm{i\Delta \Phi}(t)}$ is represented by a complex number with a unit magnitude and an angle determined by ΔΦ(t).

#### 2.5.3 Integrative analysis of SHAP and PLV for explainable EEG feature interpretation

To enhance the interpretability of the proposed Multi-Branch GAT-GRU-Transformer model, we adopt a complementary two-step framework involving SHAP and PLV analyses, bridging the gap between model decision-making and neurophysiological validation. SHAP, as an explainable AI technique, quantifies the contribution of EEG features—channels, time points, and frequency bands—to the model’s classification decisions, identifying critical elements driving the prediction of finger motor intentions. For instance, SHAP analysis highlights the dominant role of channels C3, C4, and Cz in the Beta (13–30 Hz) and Gamma (30–100 Hz) bands, aligning with their established roles in motor execution and sensory feedback. Within the interpretability framework, PLV serves as a neurophysiological validation tool that works in tandem with SHAP. While SHAP elucidates the model’s internal decision-making by quantifying feature importance, PLV not only verifies whether these decisions align with actual brain activity patterns but also contributes actively during model training. Specifically, PLV-derived phase synchrony is embedded into the GAT adjacency matrix, guiding the attention mechanism to learn spatial dependencies among EEG channels. This dual role—validation and structural guidance—enhances the model’s transparency, biological plausibility, and interpretability.

To validate whether the model’s reliance on these features corresponds to neurophysiologically meaningful patterns, we subsequently employ PLV to examine the phase synchronization between EEG channels, focusing on the same frequency bands identified by SHAP. PLV analysis reveals high synchronization between C3 and C4, as well as between C3/C4 and Cz, particularly in the Beta and Gamma bands, indicating strong functional connectivity within the motor cortex and between motor and midline regions during finger motor imagery tasks. This synchronization pattern corroborates the neurophysiological significance of the features prioritized by the model, as the motor cortex (C3 and C4) is known to play a central role in motor control, while Cz facilitates cross-regional integration.

The alignment between SHAP and PLV results suggests that the model effectively captures neurophysiologically relevant EEG features, such as the activation of the motor cortex and its functional connectivity with other brain regions, during finger motor intention tasks. For example, channels with high SHAP values (e.g., C3 and C4) correspond to regions with strong phase synchronization in PLV analysis, reflecting cooperative activity within the motor cortex that is consistent with established neuroscience findings (Miasnikova and Franz, 2022). This complementary analysis not only enhances the transparency of the model’s decision-making process but also provides a neuroscientific basis for its feature selection, thereby increasing its reliability for applications in neuroscience and brain-computer interfaces (He et al., 2025).

## 3 Results

### 3.1 Evaluation indicators

The objective of this experiment is to comprehensively evaluate the model’s performance and interpretability from multiple perspectives, including classification performance, comparison with baseline methods, interpretability analysis, and ablation studies. First, the experiment assesses the model’s classification effectiveness in the five-finger motor imagery task, utilizing accuracy and the confusion matrix as primary evaluation metrics to provide a holistic measure of the model’s recognition capability. Accuracy quantifies the overall correctness of the model’s predictions, reflecting its average classification performance across all categories, and is calculated as Equation 9:


(9) $$\begin{array}{c} A\text{ccuracy}=\frac{\mathrm{TP}+\mathrm{TN}}{\mathrm{TP}+\mathrm{TN}+\mathrm{FP}+\mathrm{FN}} \end{array}$$

Precision measures the proportion of correctly predicted positive instances out of all instances predicted as positive and is calculated as Equation 10. It reflects the model’s ability to avoid false positives. In the context of finger motor imagery, high precision indicates that when the model predicts a particular finger movement, it is likely to be correct.


(10) $$\begin{array}{c} \text{Prec}i\text{sion}=\frac{\mathrm{TP}}{\mathrm{TP}+\mathrm{FP}} \end{array}$$

Recall assesses the proportion of actual positive instances that are correctly identified by the model. It reflects the model’s capacity to detect relevant instances and is calculated as Equation 11. High recall indicates that the model can successfully capture most occurrences of a specific finger movement, minimizing false negatives.


(11) $$\begin{array}{c} \text{Recall}=\frac{\mathrm{TP}}{\mathrm{TP}+\mathrm{FN}} \end{array}$$

F1-score is the harmonic mean of precision and recall, offering a balanced metric that accounts for both false positives and false negatives as Equation 12. It is particularly useful when the class distribution is uneven or when both precision and recall are important for task performance. A high F1-score suggests that the model achieves both high detection accuracy and reliability across classes.


(12) $$\begin{array}{c} F1-\text{score}=2*\frac{\text{Precision}*\text{Recall}}{\text{Precision}+\text{Recall}} \end{array}$$

These metrics are computed for each of the five finger classes (thumb, index, middle, ring, and little fingers), enabling class-wise performance analysis and facilitating identification of specific strengths and weaknesses in the model’s predictions.

The confusion matrix offers a detailed analysis of the model’s classification performance across different categories, visually presenting the correct classification rates and misclassification patterns for each class. This facilitates the identification of model biases and easily confused categories.

### 3.2 Experimental results

To validate the superiority of the proposed model, we conduct comparative experiments against state-of-the-art deep learning models, including convolutional neural networks (CNNs), recurrent neural networks (RNNs), and recently developed EEG-specific models. These baseline models represent the cutting-edge approaches in motor intention recognition. For a fair comparison, all models are trained and tested on the same dataset while following an identical preprocessing pipeline. The evaluation metrics remain consistent with those used in the classification performance assessment. By analyzing the comparative results, we quantitatively assess the performance improvements achieved by the proposed model.

**Figure 4:**
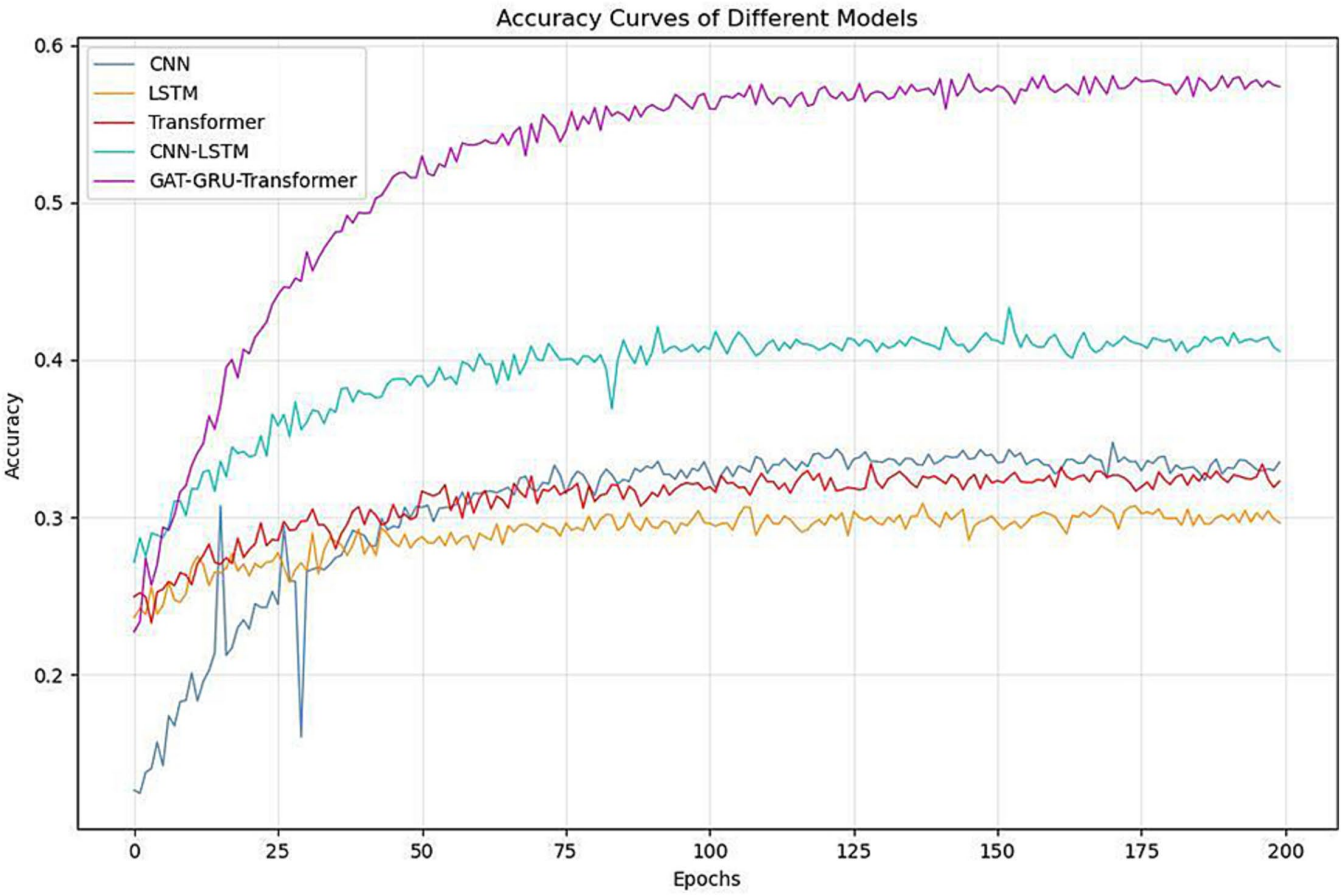
Comparison of classification accuracy between different models and multi-branch GAT-GRU-transformer model.

Figure 4 and Table 1 demonstrate the performance comparison across different models. As shown in the training curves over 200 epochs, CNN, LSTM, and Transformer models plateau around 0.43–0.46 in test accuracy, highlighting their limitations in capturing the complex spatial, temporal, and frequency dynamics of EEG signals. The CNN-LSTM hybrid offers a moderate improvement, reaching a test accuracy of 0.5032. In contrast, the proposed GAT-GRU-Transformer model shows a rapid increase in accuracy during the first 25 epochs and stabilizes around 0.5576, significantly outperforming all baseline models. Specifically, the proposed model achieves the highest training accuracy (0.6640) and test accuracy (0.5576), surpassing CNN (0.5180/0.4323), LSTM (0.5440/0.4621), Transformer (0.5190/0.4330), and CNN-LSTM (0.5980/0.5032). The performance gains are attributed to the proposed model’s architecture, which integrates Graph Attention Networks (GAT) for spatial feature extraction, GRU and Transformer for temporal dependencies, and 1D CNN for frequency characteristics. All models were trained and evaluated under identical conditions to ensure fairness. The consistent superiority of the proposed model in both learning dynamics and final classification accuracy underscores its effectiveness in decoding fine-grained finger motor imagery from EEG signals.

**Table 1:** Performance comparison of different models on the kaya EEG finger motor imagery dataset.

Model	Feature type	Architecture	Training accuracy	Test accuracy
CNN	Spatial-frequency	2D CNN	0.5180	0.4323
LSTM	Temporal	LSTM	0.5440	0.4621
Transformer	Temporal	Transformer Encoder	0.5190	0.4330
CNN-LSTM	Spatial–temporal	CNN + LSTM	0.5980	0.5032
Proposed model	Multi-modal	GAT-GRU-Transformer + 1D CNN	0.6640	0.5576

The classification results across the five fingers demonstrate noticeable variation in performance, with an overall model accuracy of 55.76% on the Kaya dataset. Among all classes, the thumb achieves the best performance, with a precision of 64.73%, recall of 65.28%, and an F1 score of 65.01%, indicating that the model effectively distinguishes its motor intentions. The middle finger follows closely, with a precision of 60.12%, recall of 58.67%, and an F1 score of 59.39%, suggesting similarly reliable classification performance.

The index finger shows moderate performance, with a precision of 54.23%, recall of 52.12%, and an F1 score of 53.17%, slightly lower than the thumb and middle finger. This may reflect some degree of overlap with adjacent classes, which we explore further through the confusion matrix analysis below. In contrast, the ring and little fingers exhibit the weakest performance, with F1 scores of 43.89% (precision: 44.56%, recall: 43.23%) and 48.33% (precision: 49.01%, recall: 47.66%), respectively as shown in Table 2. These lower scores highlight the model’s difficulty in differentiating between these two classes, potentially due to their physiological and signal-level similarity, as the ring and little fingers share overlapping neural representations in the motor cortex.

**Table 2:** Classification performance by finger class (precision, recall, and F1 score).

Class (Finger)	Precision	Recall	F1 score
Thumb	0.6473	0.6528	0.6501
Index	0.5481	0.5162	0.5317
Middle	0.6049	0.5833	0.5939
Ring	0.4264	0.4521	0.4389
Little	0.4718	0.4953	0.4833

**Figure 5:**
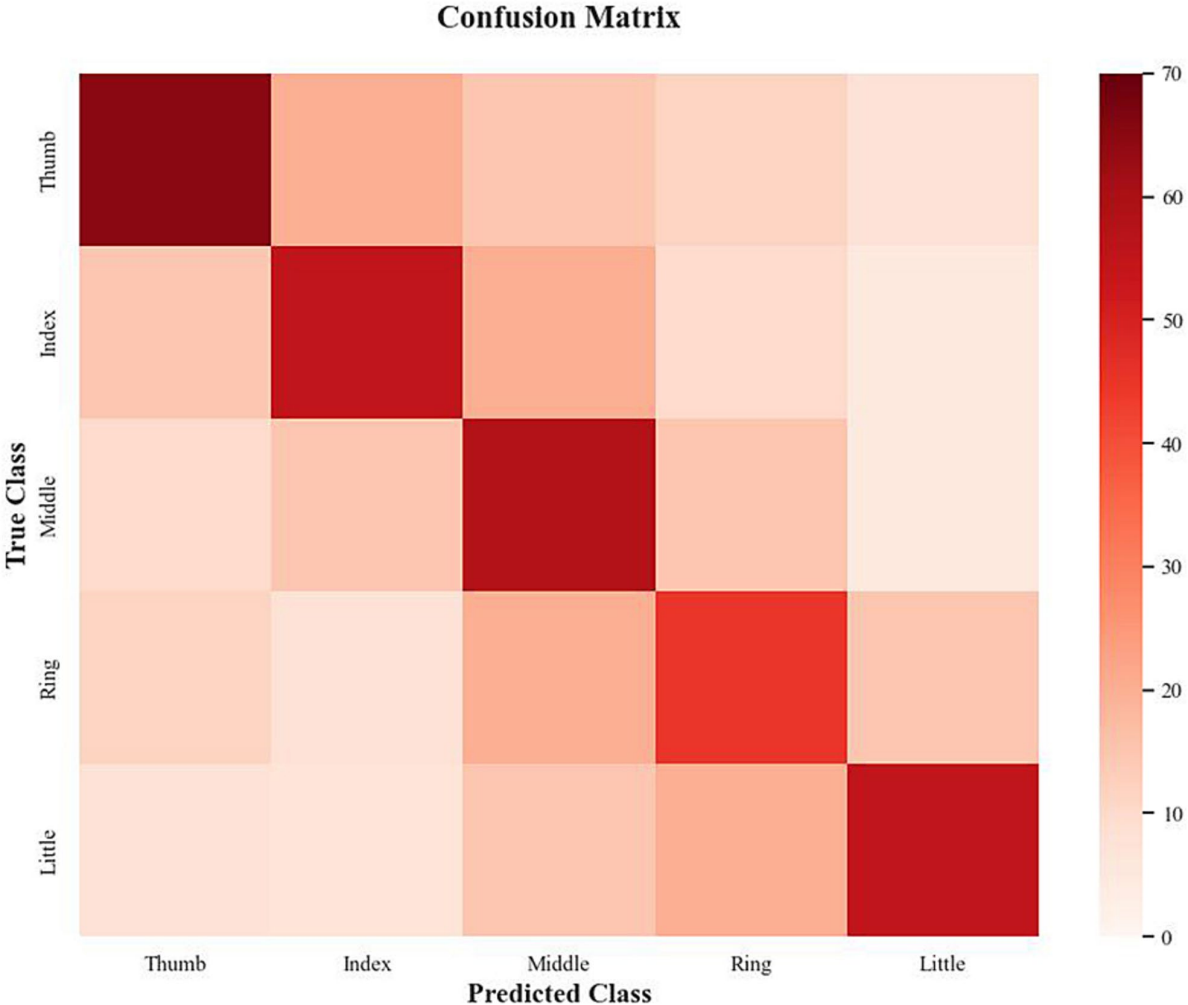
Finger classification confusion matrix.

To further investigate the model’s classification behavior, we generated a confusion matrix shown in Figure 5 to analyze misclassification patterns across the five finger classes. The confusion matrix reveals that the model achieves high discriminability for the thumb, with a true positive rate of 65.3% and a low confusion rate of <5% with other fingers, reflecting the distinct EEG patterns associated with thumb movements, likely due to stronger activation in the motor cortex (e.g., C3 and C4 channels in the Beta band). The middle finger also shows strong performance, with a true positive rate of 58.7% and minimal confusion with non-adjacent fingers (e.g., <3% confusion with the thumb). However, the index finger exhibits moderate confusion with the middle finger, with a misclassification rate of 12.4%, likely due to their adjacent positions on the hand and partially overlapping neural representations.

The most significant misclassification occurs between the ring and little fingers, with a confusion rate of 15.2%, meaning that 15.2% of ring finger trials were incorrectly classified as little finger movements, and vice versa. This high confusion rate aligns with the lower F1 scores for these classes and suggests that the subtle EEG pattern differences between the ring and little fingers—such as weaker and less distinct event-related desynchronization (ERD) in the Beta band—are challenging for the model to distinguish. Additionally, the little finger shows a 9.8% misclassification rate with the ring finger, further highlighting their signal-level similarity. Overall, these findings indicate that while the proposed multi-branch model demonstrates strong recognition capability for central fingers such as the thumb and middle finger, further refinement is required to improve the separability of the ring and little fingers.

To validate the effectiveness of the proposed Multi-Branch GAT-GRU-Transformer model, we conducted a comprehensive comparison with existing methods using the same Kaya dataset. The comparison includes four baseline methods: a traditional Support Vector Machine (SVM) with handcrafted features (Kaya et al., 2018), Filter Bank Common Spatial Pattern (FBCSP) with SVM (AsaBarthMaron, 2022), a standard Convolutional Neural Network (CNN) for EEG classification (Gifford, 2022), and EEGNet, a compact CNN specifically designed for EEG classification (Limbaga et al., 2022). These methods were selected to represent a range of approaches—from traditional feature engineering to deep learning—ensuring a fair and comprehensive evaluation.

As shown in Table 3, the proposed model achieves a peak classification accuracy of 55.76% on the Kaya dataset, surpassing the traditional SVM method by 12.76% (43.00%), the FBCSP method by 9.76% (46.00%), the CNN model by 12.46% (43.30%), and the EEGNet model by 4.03% (51.73%). This consistent improvement over all baseline methods underscores the advantages of the multi-branch architecture, which enhances EEG feature representation by integrating spatial modeling through Graph Attention Networks (GAT), temporal dynamics capture via the GRU and Transformer, and frequency-domain feature extraction using 1D CNNs. The comprehensive fusion of these multi-dimensional features enables the proposed model to effectively capture the complex spatio-temporal-frequency patterns inherent in EEG data, leading to superior classification performance compared to existing methods. In contrast, SVM, FBCSP, and the standard CNN rely on limited feature extraction techniques, which restrict their capacity to fully represent the non-stationary and noisy nature of EEG signals in the Kaya dataset. For instance, SVM uses power spectral density features, while FBCSP extracts spatial patterns across multiple frequency bands, but both methods struggle with inter-subject variability and require extensive preprocessing, resulting in lower accuracies (43.00 and 46.00%, respectively). The CNN model, with an accuracy of 43.30%, performs similarly to traditional methods, as it primarily focuses on spatial and frequency features through convolutional layers but lacks the ability to model temporal dynamics or inter-channel relationships. EEGNet, while specifically designed for EEG signal processing, achieves an accuracy of 51.73%, falling short of our model by 4.03%. Although EEGNet effectively extracts spatial and frequency features through its compact CNN architecture, it does not explicitly model long-range temporal dependencies or inter-channel relationships, aspects that our model addresses through the GRU-Transformer branch and GAT-based spatial modeling, respectively.

**Table 3:** Comparison of classification accuracy between proposed model and existing methods.

Method	Dataset	Feature type	Architecture	Accuracy
SVM (Kaya)	5F	Handcrafted	SVM Classifier	0.4323
FBCSP (AsaBarthMaron)	5F	Spatial	SVM Classifier	0.4621
CNN (Gifford)	5F	Spatial-Frequency	1D CNN	0.4330
EEGNet (Neil)	5F	Spatial-Frequency	Compact CNN	0.5173
Proposed model	5F	Multi-modal	GAT-GRU-Transformer + 1D CNN	0.5576

To statistically validate the performance difference between our proposed Multi-Branch GAT-GRU-Transformer model and the EEGNet baseline, we conducted a McNemar test on their predictions over the test dataset. The resulting contingency table is presented in Table 4. Specifically, our model correctly classified 62 instances that EEGNet misclassified, while EEGNet correctly classified 38 instances that our model misclassified. The remaining samples were classified identically by both models, with 280 correctly predicted by both and 120 misclassified by both. The McNemar test yielded a chi-square statistic of 5.76 with a *p*-value of 0.021, indicating a statistically significant performance difference in favor of our proposed model (*p* < 0.05). This suggests that the performance improvement observed is unlikely due to random variation and reflects a meaningful advancement in classification capability.

**Table 4:** McNemar test contingency table.

Model comparison	EEGNet correct	EEGNet incorrect
Proposed model correct	a = 280	b = 62
Proposed model incorrect	c = 38	d = 120

Beyond performance, the proposed model offers significant advantages in interpretability, further distinguishing it from the baseline methods. By incorporating SHAPand PLV analyses, our model provides neuroscientific insights into its decision-making process, such as the reliance on C3, C4, and Cz channels in the Beta and Gamma bands, which align with strong functional connectivity patterns validated by PLV. These interpretability features are absent in SVM, FBCSP, CNN, and EEGNet, making our model more suitable for applications requiring both high performance and neurophysiological understanding, such as brain-computer interfaces and neuroscience research. These findings strongly confirm the effectiveness and innovativeness of the proposed Multi-Branch GAT-GRU-Transformer model in EEG signal decoding tasks, demonstrating its superiority over existing techniques on the Kaya dataset.

### 3.3 Ablation experiments

To systematically evaluate the contribution of each module to the overall performance of the model, an ablation study was conducted using a controlled variable method. By gradually removing the core components of the model, the role of each module in the feature extraction process was quantitatively analyzed, thereby verifying the effectiveness of the model architecture design. Three sets of control models were constructed for this ablation study, focusing on spatial modeling, temporal modeling, and frequency feature extraction, respectively.

In the spatial modeling ablation study, the objective was to quantify the contribution of spatial relationship modeling to EEG signal classification. Specifically, the spatial topology modeling branch constructed using the Graph Attention Network (GAT) was removed, while retaining the temporal modeling module (GRU + Transformer) and the frequency feature extraction module (1D CNN). This configuration was designed to validate the effectiveness of spatial correlation features among EEG channels in the classification task.

In the temporal modeling ablation study, the aim was to assess the critical role of the Transformer in modeling long-term temporal dependencies. In this setup, the Transformer-based temporal modeling module was removed, leaving only the GRU unit for temporal feature extraction. By comparing this setup with the complete model, the study explored the advantages of the Transformer network in decoding the dynamic properties of EEG signals over time.

In the frequency feature ablation study, the goal was to evaluate the influence of frequency-domain information on classification performance. The 1D CNN-based frequency feature extraction branch was removed, while retaining the combined spatio-temporal modeling modules (GAT + GRU + Transformer). This experimental configuration was designed to reveal the importance of EEG spectral features in pattern recognition.

Under consistent hyperparameter settings, all ablation models were trained and tested on the same dataset. Accuracy was used as the primary evaluation metrics as shown in Table 5. The results demonstrated that the complete model achieved an accuracy of 55.7%, which was significantly higher than the performance of the ablation variants. This confirms the existence of a complementary and enhancing effect among the different modules.

**Table 5:** Results of ablation experiments.

Model	Accuracy
Complete model	0.5576
Removal of GAT branch	0.4867
Removal of transformer	0.4991
Removal of 1D CNN branch	0.5142

Ablating the spatial modeling module resulted in an accuracy drop of approximately 7.1% points (from 55.7 to 48.6%), indicating that the GAT network plays a crucial role in capturing the spatial topological relationships among EEG channels. Through the graph attention mechanism, the model can dynamically learn spatial interactions between electrodes, thereby extracting features related to the coordinated activity of different brain regions. The contribution of spatial features to the overall performance of the model is significant, especially in motor imagery tasks involving inter-regional interactions, where the spatial information modeled by the GAT enhances the model’s discriminative ability.

Ablating the temporal modeling module led to an accuracy drop of approximately 5.8% points (from 55.7 to 49.9%), highlighting the prominent advantage of the Transformer in modeling long-term temporal dependencies. Compared with the GRU’s capability of local temporal modeling, the Transformer, through the self-attention mechanism, captures dynamic changes in EEG signals on a global scale. This advantage is particularly evident when processing event-related potentials and temporal synchronization patterns across different time periods, where the Transformer demonstrates superior modeling ability.

Ablating the frequency feature extraction module resulted in an accuracy loss of approximately 4.3% points (from 55.7 to 51.4%), underscoring the importance of 1D CNN in extracting frequency-domain information such as Beta and Alpha rhythms. The time-frequency representations extracted by CNN reveal rhythm patterns associated with motor intentions, providing crucial supplementary information for the classification task. Therefore, the combined modeling of frequency features with spatial and temporal features is a key factor in enhancing classification performance.

The experimental results reveal a significant complementary and enhancing mechanism among the different modules, confirming that joint modeling of spatial, temporal, and frequency features is indispensable in EEG classification tasks. The spatial modeling module, implemented using the GAT network, dynamically models spatial relationships between EEG channels through adaptive attention weights, overcoming the limitations of traditional fixed adjacency matrices. The GAT network is capable of automatically learning the connection weights between these key channels, thereby improving the model’s adaptability and classification accuracy in different finger motor imagery tasks.

The temporal modeling module, consisting of GRU and Transformer, captures the dynamic evolution of EEG signals over time. The GRU effectively models short-term temporal dependencies through its gating mechanism, enabling the model to capture dynamic changes within short time windows. However, EEG signals often exhibit long-term temporal correlations, especially in ERPs and rhythmic patterns. The Transformer, with its self-attention mechanism, demonstrates significant advantages in modeling long-range temporal dependencies. Unlike the GRU’s local modeling approach, the Transformer simultaneously attends to global time steps, identifying dynamic evolution trends across different time segments. The experimental results show that the Transformer’s strength in modeling long-term dependencies is directly reflected in enhanced classification performance in complex motor tasks.

The frequency analysis module, implemented using 1D CNN, extracts EEG features across different frequency bands, including Delta, Theta, Alpha, Beta, and Gamma. Beta rhythms (13–30 Hz) are most strongly associated with motor control and sensory feedback, playing a key role in finger movement. Alpha rhythms (8–13 Hz) are linked to motor inhibition and attentional focus, while Gamma rhythms (30–40 Hz) are associated with sensorimotor integration and higher cognitive processes. Through multi-band modeling, CNN effectively captures rhythm patterns related to motor intentions, thereby improving the model’s discriminative ability. Moreover, the frequency features complement the spatial and temporal features, with the time-frequency patterns extracted by CNN providing distinct classification information aligned with physiological signals.

The multi-modal fusion mechanism enables a complementary and orthogonal modeling strategy within the feature space. The GAT network focuses on the spatial structure of EEG signals, capturing dynamic connectivity between channels. The Transformer enhances the model’s capacity for long-term temporal dependency modeling, strengthening the combination of spatial and frequency features. The 1D CNN extracts rhythm patterns from different frequency bands, providing physiologically consistent discriminative features that complement the spatio-temporal modeling results. The spatial modeling (GAT) enhances the integrity of input features for temporal modeling (Transformer) and frequency modeling (CNN). The temporal modeling (Transformer) provides stronger dynamic modeling capacity in complex motor tasks, enhancing the combined effect of spatial and frequency features. The frequency modeling (CNN) offers rhythm-based discriminative features consistent with neural signals, which complement the spatial and temporal modeling results, further improving overall model performance.

## 4 Discussion

### 4.1 SHAP-based analysis of EEG features

To gain deeper insights into the decision-making mechanism of the model, we employ the SHAP framework. Specifically, DeepExplainer is used to compute SHAP values for each EEG channel and different frequency band features, quantifying their contributions to classification decisions. A higher SHAP value indicates a greater positive influence of a feature on the model’s classification outcome (Ameen et al., 2024).

**Figure 6:**
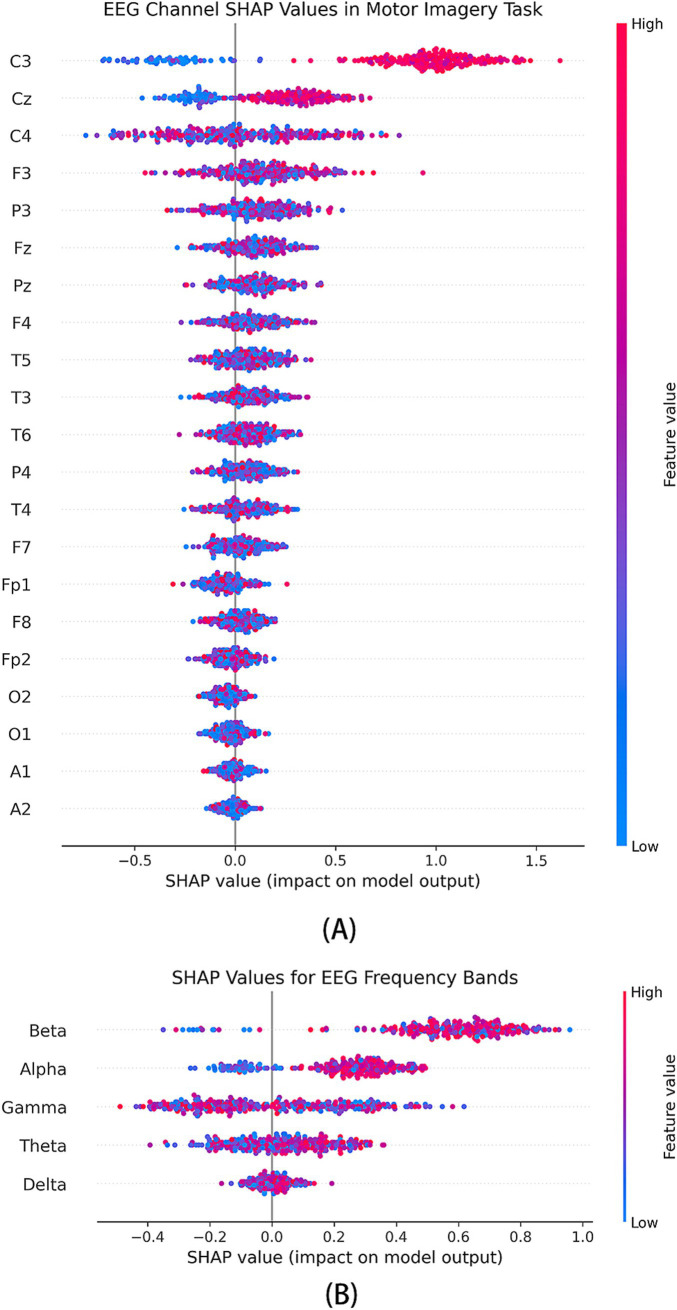
SHAP analysis of feature contributions in the multi-branch GAT-GRU-transformer model. **(A)** SHAP summary plot for EEG channels, showing high contributions from C3, C4, and Cz. **(B)** SHAP summary plot for frequency bands, highlighting the importance of Beta and Gamma bands.

The Figure 6A illustrates the SHAP value distribution across all EEG channels. The results indicate that channels C3, Cz, and C4 exhibit significant SHAP value variations across different classes, suggesting their critical role in the model’s decision-making process. This finding aligns with existing neurophysiological studies, which have established that C3 and C4 correspond to the primary motor cortex (M1), a key region involved in limb movement control. Specifically, the C3 channel, associated with the left primary motor cortex, shows a substantial positive SHAP contribution in right-hand finger movement tasks, reinforcing the dominant role of the left hemisphere in right-hand motor control. In contrast, the C4 channel, positioned over the right primary motor cortex, primarily controls the left hand but still exhibits moderate SHAP values, potentially reflecting bilateral cortical cooperation during motor execution. Meanwhile, the Cz channel, located over the central sulcus, demonstrates notable SHAP contributions, suggesting that the model integrates midline cortical activity to capture sensorimotor coordination during finger movement tasks.

Additionally, FP1 and FP2 channels show distinct SHAP contributions in certain classes, which may be attributed to the prefrontal cortex’s involvement in motor planning and attentional control. The overall SHAP value distribution strongly aligns with the functional organization of motor-related brain regions, reinforcing the biological plausibility of the model in effectively extracting motor-relevant EEG features.

In the SHAP analysis, we visualize the distribution of SHAP values across different frequency bands as shown in Figure 6B. The x-axis represents SHAP value magnitudes, while the y-axis denotes various EEG frequency bands and channel features. Each data point is color-coded according to feature magnitude, with red indicating higher values and blue representing lower values. The results reveal that the Beta (13–30 Hz) and Gamma (30–100 Hz) bands exhibit the most significant positive contributions to the model, whereas the contributions of Delta and Theta bands are relatively lower. Beta Band Closely associated with motor execution and sensory feedback, Beta activity typically exhibits transient synchronization and desynchronization during movement preparation and execution. In this study, Beta-band SHAP values show significant positive contributions at the C3 and C4 channels, suggesting that the model may rely on Beta activity to capture sensorimotor feedback signals during motor execution. Gamma Band essential for attentional modulation and inter-cortical communication in sensorimotor tasks (Liu et al., 2024) SHAP value distribution in this study indicates that Gamma activity contributes most prominently at the C3, C4, and Cz channels, implying that the model leverages high-frequency neural oscillations to extract fine motor control information. Alpha Band primarily related to attention and sensorimotor rhythms. A notable positive contribution is observed at the Cz channel, potentially reflecting the role of Alpha activity in sensory feedback processing during motor imagery tasks. Theta and Delta Bands exhibit lower SHAP contributions, suggesting that in the context of finger movement tasks, mid-to-high frequency features are more critical than low-frequency components.

Overall, the SHAP analysis highlights the dominance of Beta and Gamma bands in the model’s classification decisions, reinforcing their relevance in motor execution and sensorimotor processing. The observed frequency-specific contributions provide valuable insights into the neurophysiological basis of EEG-based motor intention recognition.

### 4.2 PLV analysis for functional connectivity

To further investigate the functional connectivity patterns between different brain regions, we computed the Phase Locking Value (PLV) for all pairs of EEG channels to quantify phase synchronization between them. A higher PLV value indicates stronger synchronization, suggesting potential cooperative activity between these brain regions during the finger movement task (Wang et al., 2020).

**Figure 7:**
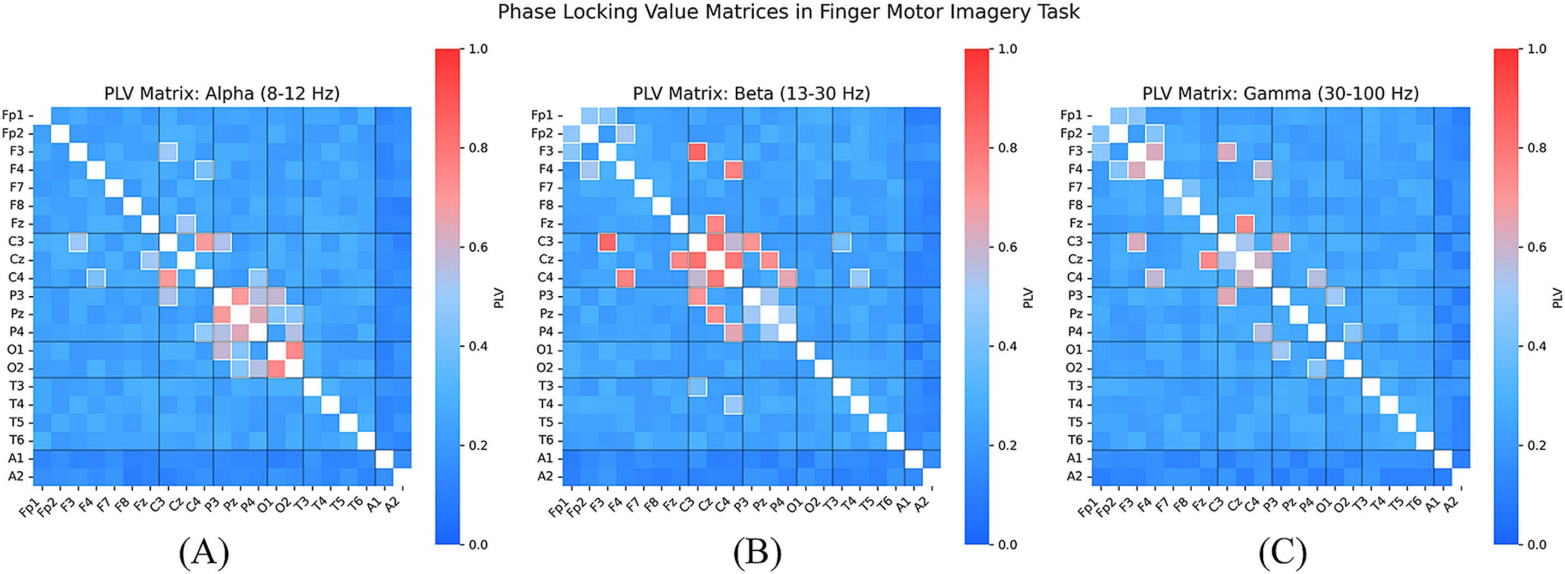
PLV connectivity matrices in the Alpha **(A)**, Beta **(B)**, and Gamma **(C)** bands, revealing strong synchronization between C3, C4, and Cz, thereby validating the neurophysiological relevance of the model’s prioritized EEG features.

We visualized the PLV heatmap for different EEG channel pairs as shown in Figure 7, where the horizontal and vertical axes represent distinct EEG channels, and the color intensity indicates the magnitude of the PLV value. The results reveal that regions exhibiting high synchronization are predominantly observed in the Beta frequency band, with strong functional connectivity between the central region (C3, C4) and the parietal region (Pz), suggesting their critical involvement in the finger movement task. Notably, the PLV values between C3 and C4 remain consistently high across different finger movement tasks, indicating the potential role of bilateral coordination within the primary motor cortex in finger movement control. In Figure 7, we present the PLV matrices across the Alpha, Beta, and Gamma frequency bands. The results indicate that in the Alpha band, Cz and C3/C4 exhibit strong phase synchronization, which may be associated with attentional modulation and sensory feedback during motor tasks. In the Beta band, the PLV between C3 and C4 significantly increases, reinforcing the crucial role of interactions within the primary motor cortex during motor execution. Enhanced Gamma band synchronization is observed across multiple channel pairs, particularly between C3-Cz and C4-Cz, suggesting that high-frequency oscillations may contribute to interregional information integration and rapid motor adjustments. These findings provide neurophysiological support for the SHAP analysis, which highlights the significance of these channels in model decision-making (Jian et al., 2017).

### 4.3 Joint SHAP-PLV analysis and neural mechanisms

To further explore the relationship between the learned model features and underlying neural mechanisms, we conducted a joint interpretation of SHAP and PLV results. The SHAP analysis identifies the key frequency-domain features and EEG channels that the model relies on for finger movement classification, while the PLV analysis reveals intercortical functional connectivity patterns across different frequency bands. Therefore, this joint analysis provides valuable insights into the neurophysiological mechanisms leveraged by the model during learning.

In the Beta and Gamma bands, SHAP analysis indicates a significantly increased importance of C3 and C4 channels in model decision-making, which aligns with the high synchronization observed between these channels in PLV analysis. The enhanced synchronization in Beta and Gamma bands may reflect information exchange and dynamic coordination between the primary motor cortices during finger movement tasks (Xu et al., 2021). This suggests that the model may optimize classification performance by capturing phase synchronization features within the sensorimotor cortex.

In the Alpha band, SHAP analysis reveals a marked increase in the importance of the Cz channel, consistent with the high synchronization observed between Cz and C3/C4 in PLV analysis (Sylvester et al., 2024). This finding suggests that Alpha-band synchronization plays a critical role in sensory feedback and motor planning.

Moreover, the spatial distribution of SHAP values exhibits consistency with the spatial patterns of synchronization in the PLV heatmap. For instance, the high SHAP contributions near the central region (Cz) and the primary motor cortex (C3, C4) closely correspond to the high synchronization observed in the same regions in PLV analysis. Notably, the consistency between SHAP and PLV values in Beta and Gamma bands further supports the biological plausibility of the model’s ability to extract features related to motor execution and sensory feedback (Hosseini and Shalchyan, 2022) is finding reinforces the hypothesis that the model achieves high classification performance by capturing dynamic phase synchronization features within the sensorimotor cortex, thereby enhancing its ability to distinguish different finger movement tasks (Tian et al., 2025).

In conclusion, PLV analysis reveals the synchronization characteristics of intercortical communication across different frequency bands, while SHAP analysis confirms the importance of these features in model classification decisions. This joint analytical approach provides strong theoretical support for understanding the neural mechanisms underlying finger movements and contributes to the optimization of brain-computer interface models.

## 5 Conclusion and future work

This study presents a novel multi-branch GAT-GRU-Transformer model for EEG-based classification of five-finger motor imagery, achieving a classification accuracy of 55.76% on the Kaya dataset. Although the overall accuracy remains moderate, it represents a notable improvement compared to baseline methods such as SVM (43.00%), FBCSP (46.00%), CNN (43.30%), and EEGNet (51.73%). The proposed architecture integrates Graph Attention Networks (GAT) for spatial feature extraction, GRU-Transformer modules for modeling temporal dependencies, and one-dimensional Convolutional Neural Networks (1D CNN) for capturing frequency-domain information. This multi-branch design effectively models the complex spatio-temporal-frequency dynamics of EEG signals.

Beyond performance improvements, the model incorporates interpretability through SHAP and phase-locking value (PLV) analyses. These analyses reveal that the model predominantly relies on EEG activity in the C3, C4, and Cz channels within the Beta and Gamma frequency bands—regions and rhythms well-established in motor imagery research. This alignment with known neurophysiological patterns not only enhances the transparency of the model’s decision-making process but also provides meaningful neuroscientific insights into the neural correlates of fine motor intention.

While the current accuracy reflects solid performance, further improvements are necessary particularly in distinguishing more challenging classes such as the little finger. Future research directions include the application of data augmentation techniques, such as Generative Adversarial Networks (GANs), to synthesize EEG signals and mitigate class imbalance and inter-subject variability. Advanced regularization strategies and transfer learning approaches, including spatial dropout and pretraining on large-scale EEG datasets, will be explored to improve generalization and reduce overfitting. In addition, incorporating alternative feature modalities such as time-frequency representations may enrich the input space, while ensemble learning and systematic hyperparameter optimization are expected to further enhance classification performance.

By combining robust classification performance with interpretability, this work contributes to both brain-computer interface development and neuroscience research. Unlike conventional BCI systems that prioritize accuracy at the expense of transparency, our model delivers reliable decoding of fine-grained finger motor imagery while shedding light on the underlying neural mechanisms. In clinical contexts, the ability to decode individual finger movements offers promising potential for motor rehabilitation in patients with stroke or spinal cord injuries, supporting neurofeedback-based interventions and targeted motor imagery training. The interpretability framework also enables the design of personalized rehabilitation protocols based on individual neural patterns.

From a research standpoint, this work provides a methodological foundation for further investigations into the neural dynamics of motor imagery. Future studies may leverage larger datasets or more sophisticated connectivity metrics to deepen our understanding of brain network behavior during imagined movement. Overall, the proposed model advances the synergy between BCI technology and neuroscience, contributing to both applied and theoretical progress in the field.

## Data Availability

Publicly available datasets were analyzed in this study. This data can be found at: https://figshare.com/collections/A_large_electroencephalographic_motor_imagery_dataset_for_electroencephalographic_brain_computer_interfaces/3917698.
